# Supplementary material for: JNK signaling in pioneer neurons organizes ventral nerve cord architecture in Drosophila embryos
Source: Nat Commun. 2023 Feb 7;14:675. doi: 10.1038/s41467-023-36388-1 (PMC9905486; doi:10.1038/s41467-023-36388-1)
Supplement: Supplementary file 1 — Supplementary Information [file 41467_2023_36388_MOESM1_ESM.pdf]

JNK signaling in pioneer neurons organizes ventral nerve cord architecture in *Drosophila* embryos

Katerina Karkali[1,2,3], Timothy E. Saunders[2,4], George Panayotou[3] and Enrique Martín-Blanco[1,2*]

1. Instituto de Biología Molecular de Barcelona (CSIC), Parc Cientific de Barcelona, Baldiri Reixac 10-12, 08028 Barcelona, Spain

2. Mechanobiology Institute and Department of Biological Sciences, 5 Engineering Drive 1, National University of Singapore, 117411 Singapore

3. BSRC Alexander Fleming, 34 Fleming Street, 16672 Vari, Greece

4. Division of Biomedical Sciences, Warwick Medical School, University of Warwick, Coventry CV4 7AL, UK.

* Correspondence to: embbmc@ibmb.csic.es

SUPPLEMENTARY FIGURES

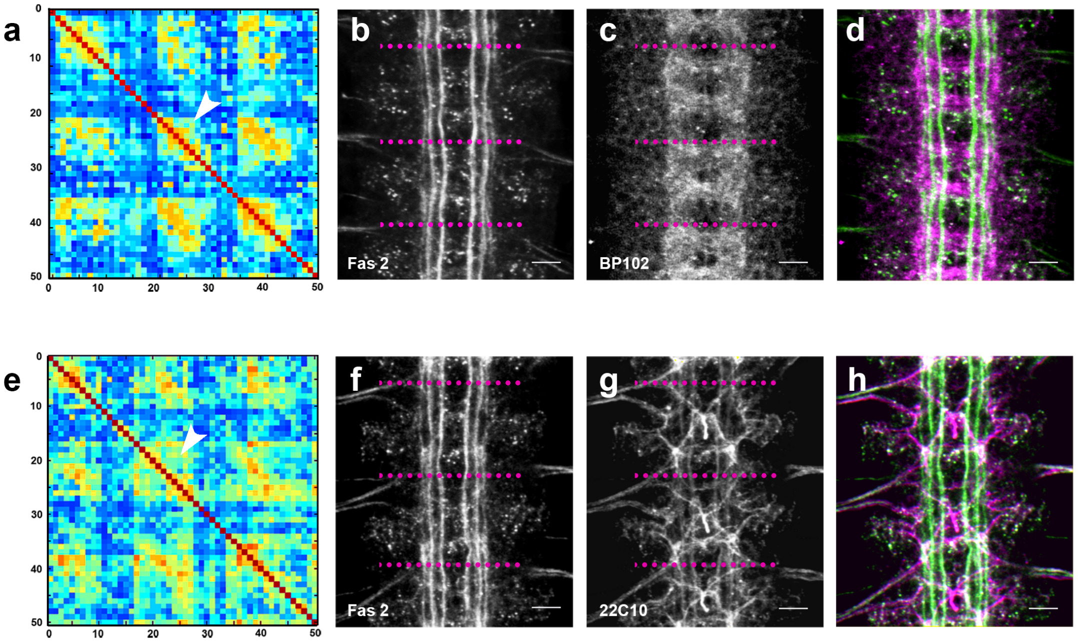
a
b
c
d
e
f
g
h
Fas 2
BP102
Fas 2
22C10

Supplementary Figure 1. Fine localization of robust 3D nodes

a) Self cross-correlation matrix of the Z sections of the image in (b). Scales and colormap as in Figure 1. A conspicuous node of robust correlation is highlighted (white arrowhead).

b to d) Images for Fas 2 ((b) - green in (d)), BP102 ((c) - magenta in (d)) and their superimposed image (d) of a double-stained 16 stage wild type embryo. Discontinued magenta lines indicate the localization of the 3D nodes in relation to the VNC pattern. Scale bar 10 µm.

e) Self cross-correlation matrix of the Z sections of the image in (f). Scales and colormap as in Figure 1. A conspicuous node of robust correlation is highlighted (white arrowhead).

f to h) Images for Fas 2 ((f) - green in (h)), 22C10 ((g) - magenta in (h)) and their superimposed image (h) of a double-stained 16 stage wild type embryo. Discontinued magenta lines indicate the localization of the 3D nodes in relation to the VNC pattern. Scale bar 10 µm.

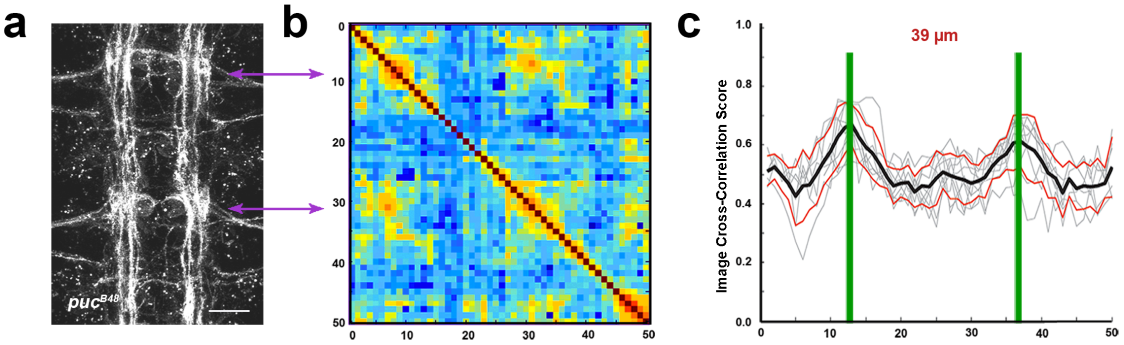
a
b
c
39 µm
Image Cross-Correlation Score

Supplementary Figure 2. Robustness and modularity of the VNC architecture in *puc*^{B48} embryos

a) Maximum projection of the VNC of a *Drosophila* 16-stage *puc*^{B48} embryo stained with Fas 2. Defasciculation and collapse of longitudinal connectives is widely spread. Scale bar 10 µm.

b) Self cross-correlation matrix of (a). Scales and colormap as in Figure 1. In *puc*^{B48}, single 3D nodes per segment are consistently developed (purple double headed arrows).

c) Image cross-correlation score along the AP axis for (b) (n = 8 embryos). Segmental Fas 2 3D nodes are on average at 39 µm from each other in *puc*^{B48} and the internodal profile flattens. The grey lines show individual profiles. The black line corresponds to Average values and the red lines to SD. The green bars indicate the positions of maximum correlation (3D nodes).

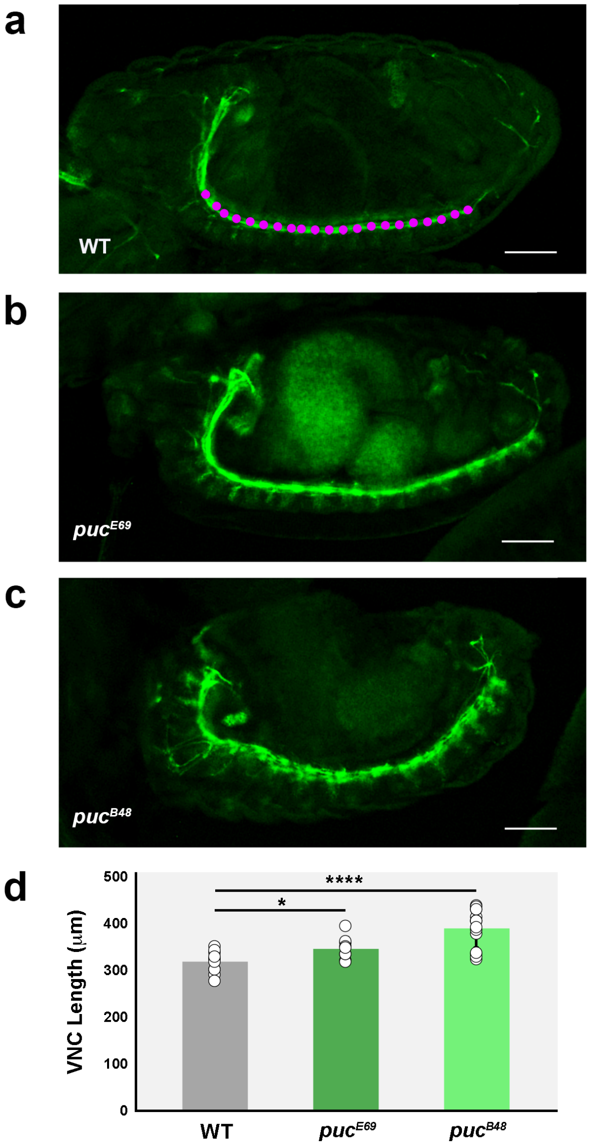
a
WT
b
pucE69
c
pucB48
d
500
400
300
200
100
0
VNC Length (μm)
****
*
WT
pucE69
pucB48

Supplementary Figure 3. Loss of VNC condensation in *puc* mutants

a) wild type, b) puc^{E69} and c) puc^{B48} embryos Fas 2 immunoreactivity. Lateral view of whole mounts of stage 17 embryos. Direct measurements of the VNC length were performed along the outline of the abdominal segments of the VNC as shown in (a) (dotted magenta line). Scale bar 10 μm.

d) VNC length in μm. Significant differences in length were detected between the wild type and *puc* alleles (wild type, (grey) n = 11; puc^{E69}, (green) n = 9; puc^{B48}, (light green) n = 15). Data are presented as mean ± SD. Parametric Student t-tests were employed. *, p = 0.0135 for puc^{E69} and ****, p < 0.0001 for puc^{B48}.

Source data are provided as a Source Data file.

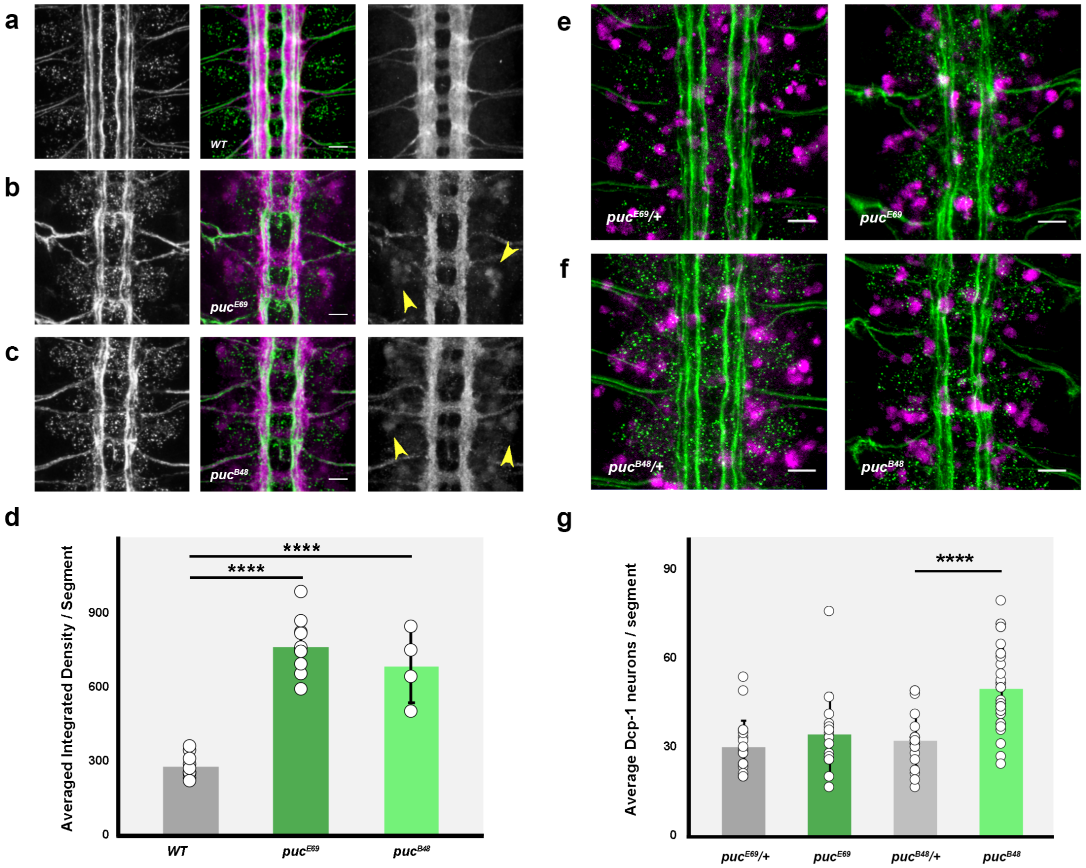
a
b
c
d
e
f
g
WT
pucE69
pucB48
pucE69/+
pucB48/+
****
****
****
Averaged Integrated Density / Segment
900
600
300
0
Average Dcp-1 neurons / segment
90
60
30
0
WT
pucE69
pucB48
pucE69/+
pucE69
pucB48/+
pucB48

Supplementary Figure 4. JNK activity and cell death in *puc* mutants

a) Single (left and right) and combined (center) channels highlighting the expression of Fas 2 (left - green) and anti-P-JNK (right- magenta) of a stage 17 wild type embryo. Scale bar 10 µm.

b) Equivalent as (a) for a stage 17 puc^{E69} embryo. Scale bar 10 µm.

c) Equivalent as (a) for a stage 17 puc^{B48} embryo. Scale bar 10 µm.

In mutant embryos ((b) and (c)) a significant fraction of the P-JNK signal remained in or around cell bodies (yellow arrowheads) and is not just found in axons, as in wild type.

d) Graph displaying the averaged Integrated Density per segment for the P-JNK signal (wild type, (grey) n = 10; puc^{E69}, (green) n = 11; puc^{B48}, (light green) n = 4). Data are presented as mean ± SD. Parametric Student t-tests were employed. Significant differences in P-JNK levels were detected between the wild type and both mutant conditions (****, $p < 0.0001$, for puc^{E69} and puc^{B48}).

e) From left to right, stage 17 heterozygous and homozygous puc^{E69} embryo stained for Fas 2 (green) and Dcp1 (magenta), which highlights those cells in the process of apoptosis. Scale bar 10 µm.

f) From left to right, equivalent images as in (e) for the puc^{B48} allele. Scale bar 10 µm.

g) Graph displaying the average number of Dcp1 positive cells per segment. puc^{E69}/+, (grey) n = 20; puc^{E69}, (green) n = 15; puc^{B48}/+, (grey) n = 24; puc^{B48}, (light green) n = 33. Data are presented as mean ± SD. Parametric Student t-tests were employed. Significant differences (****, $p < 0.0001$) were detected between puc^{B48}/+and puc^{B48} embryos. The Dcp1 positive cells number of puc^{E69} embryos appear to be larger than in the puc^{E69}/+, but this difference was not statistically significant ($p = 0.2823$).

Source data are provided as a Source Data file.

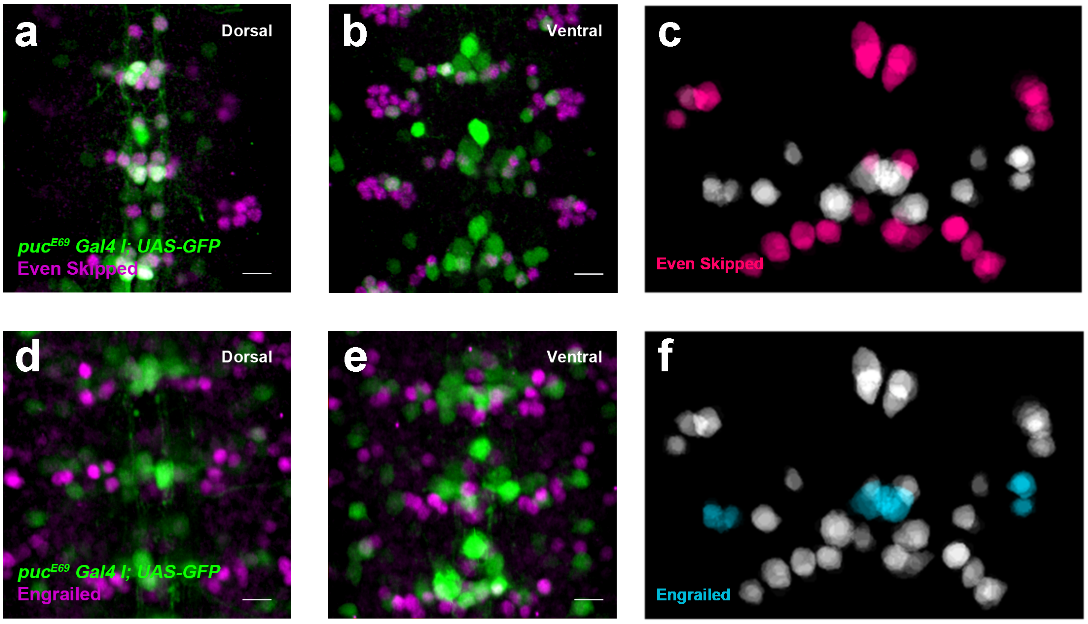
a
Dorsal
puc^{E69} Gal4 I; UAS-GFP
Even Skipped
b
Ventral
c
Even Skipped
d
Dorsal
puc^{E69} Gal4 I; UAS-GFP
Engrailed
e
Ventral
f
Engrailed

Supplementary Figure 5. Identities of *puc*-expressing neurons

a and b) Dorsal and ventral sections highlighting the expression of *puc* (green) in the VNC of stage 17 *puc*^{E69}*I-Gal4>UAS-GFP* embryo double-stained for Eve (magenta). Representative image (n = 10). Scale bar 10 µm.

c) Cartoon indicating those *puc* expressing cells also expressing Eve [magenta - aCC, pCC, RP2, U1-U3 (plus weak/variable expression in U4-U5) and 3 out of the 8 to 10 EL interneurons].

d and e) Dorsal and ventral sections highlighting the expression of *puc* (green) in the VNC of stage 17 *puc*^{E69}*I-Gal4>UAS-GFP* embryo double-stained for En (magenta). Representative image (n = 12). Scale bar 10 µm.

f) Cartoon indicating those *puc*-expressing cells also expressing En (cyan - NL1, NL2, and iVU. M5 neurons).

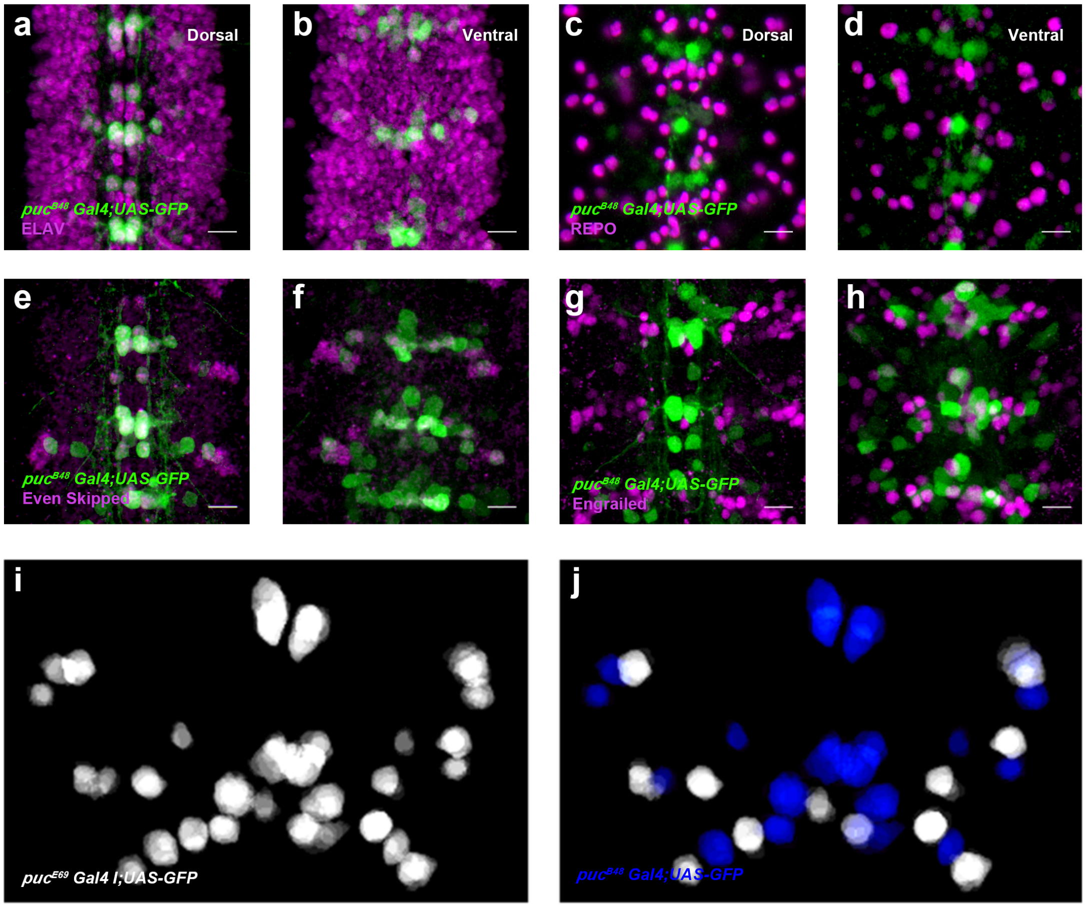
a
Dorsal
pucB48 Gal4;UAS-GFP
ELAV
b
Ventral
c
Dorsal
pucB48 Gal4;UAS-GFP
REPO
d
Ventral
e
pucB48 Gal4;UAS-GFP
Even Skipped
f
g
pucB48 Gal4;UAS-GFP
Engrailed
h
i
pucE69 Gal4 I;UAS-GFP
j
pucB48 Gal4;UAS-GFP

Supplementary Figure 6. *puc*^{B48} expressing neurons

a to d) Dorsal and ventral sections highlighting the expression of *puc* (green) in the VNC of stage 17 *puc*^{B48}*-Gal4>UAS-GFP* embryo double-stained for the pan-neuronal marker Elav ((a) and (b) - magenta) or the glial marker Repo ((c) and (d) - magenta). *puc* is expressed in a subset of neurons in each segment but not in glia. Representative image (n = 9). Scale bar 10 µm.

e to h) Dorsal and ventral sections highlighting the expression of *puc* (green) in the VNC of stage 17 *puc*^{B48}*-Gal4>UAS-GFP* embryo double-stained for Eve ((e) and (f) - magenta) or En ((g) and (h) - magenta). Representative image (n = 10). Scale bar 10 µm.

i) Cartoon representing the full set of *puc*^{E69} expressing neurons per segment in the VNC.

j) Cartoon representing the full *puc*^{E69} neurons pattern highlighting the cells expressing *puc*^{B48} (deep blue).

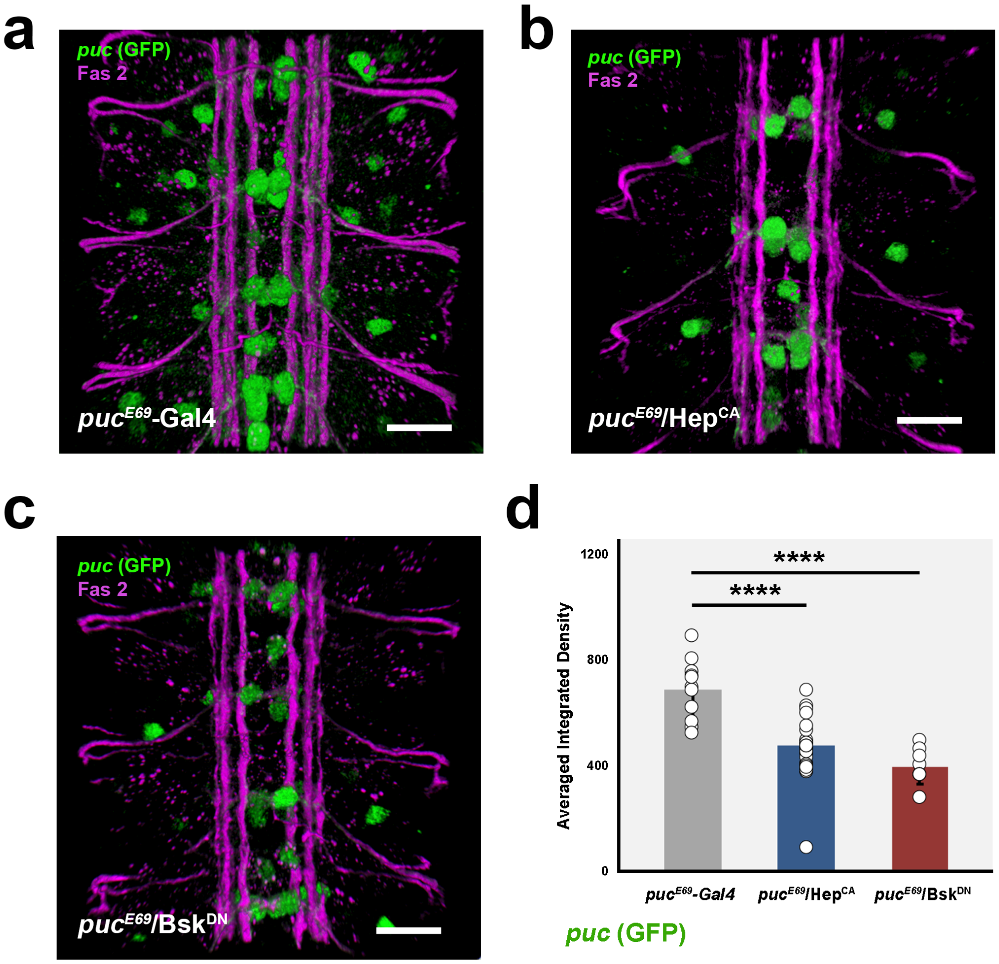
a
puc (GFP)
Fas 2
pucE69-Gal4
b
puc (GFP)
Fas 2
pucE69/HepCA
c
puc (GFP)
Fas 2
pucE69/BskDN
d
Averaged Integrated Density
1200
800
400
0
****
****
pucE69-Gal4
pucE69/HepCA
pucE69/BskDN
puc (GFP)

Supplementary Figure 7. *puc* expression is under the control of the JNK pathway

a) *puc^{E69}-Gal4 I>UAS-GFP*, b) *puc^{E69}-Gal4 I>UAS-GFP::UAS-Hep^{CA}* and c) *puc^{E69}-Gal4 I>UAS-GFP::UAS-Bsk^{DN}* stage 16 embryos double-stained for Fas 2 (magenta) and GFP (green). Scale bar 10 μm.

d) Graph displaying the averaged *puc* (GFP) Integrated Density per segment for (a) to (c). *puc^{E69}-Gal4 I*, (grey) n = 11; *puc^{E69}-Gal4 I>Hep^{CA}*, (blue) n = 24; *puc^{E69}-Gal4 I>Bsk^{DN}*, (red) n = 8. Data are presented as mean values ± SD. Parametric Student t-tests were employed. Statistically significant differences (****, $p < 0.0001$) were detected between wild type and both JNK gain and loss of function conditions.

Source data are provided as a Source Data file.

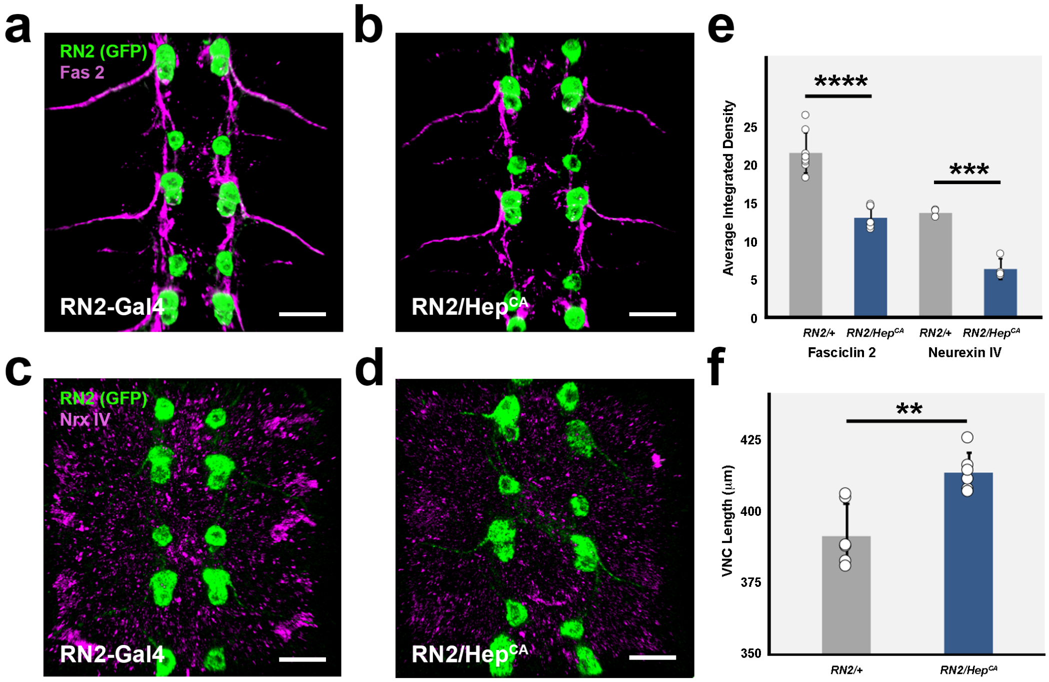
a
RN2 (GFP)
Fas 2
RN2-Gal4
b
RN2/Hep^CA
e
****
***
Average Integrated Density
25
20
15
10
5
0
RN2/+
RN2/Hep^CA
RN2/+
RN2/Hep^CA
Fasciclin 2
Neurexin IV
c
RN2 (GFP)
Nrx IV
RN2-Gal4
d
RN2/Hep^CA
f
**
VNC Length (μm)
425
400
375
350
RN2/+
RN2/Hep^CA

Supplementary Figure 8. JNK signaling modulates Fasciclin 2 and Neurexin IV expression from early developmental stages

a and c) *RN2-Gal4>UAS-GFP* and b and d) *RN2-Gal4>UAS-GFP::UAS-Hep*^{CA} stage 13 embryos double-stained for Fas 2 ((a) and (b)) or Nrx IV ((c) and (d)) (magenta) and GFP (green). Scale bar 10 µm.

e) Graph displaying the averaged Fas 2 and Nrx IV Integrated Density per segment for (a) to (d). *RN2-Gal4*, (grey) n = 8 for Fas 2, n = 3 for Nrx IV; *RN2-Gal4>Hep*^{CA}, (blue) n = 7 for Fas 2, n = 4 for Nrx IV. Data are presented as mean values ± SD. Parametric Student t-tests were employed. Statistically significant differences (****, $p < 0.001$ for Fas 2 and ***, $p = 0.0003$ for Nrx IV) were found between wild type and JNK gain of function conditions.

f) Quantification of the VNC length in µm for each condition at stage 13. *RN2-Gal4*, n = 6; *RN2-Gal4>Hep*^{CA}, n = 6. Data are presented as mean values ± SD Parametric Student t-tests were employed. Statistically significant differences in length were detected between the wild type and upon hyperactivation of JNK (**, $p = 0.0019$).

Source data are provided as a Source Data file.

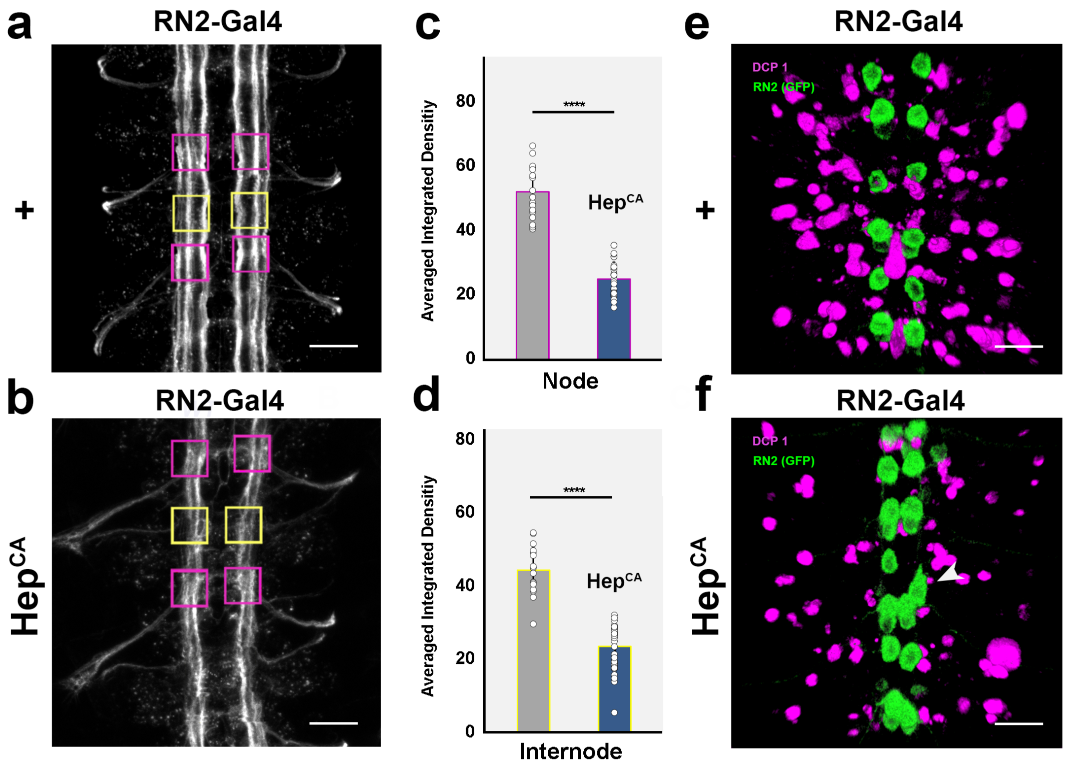
a
RN2-Gal4
+
b
RN2-Gal4
Hep^CA
c
Averaged Integrated Densitiy
80
60
40
20
0
****
Hep^CA
Node
d
Averaged Integrated Densitiy
80
60
40
20
0
****
Hep^CA
Internode
e
RN2-Gal4
DCP 1
RN2 (GFP)
+
f
RN2-Gal4
DCP 1
RN2 (GFP)
Hep^CA

Supplementary Figure 9. JNK signaling modulates Fasciclin 2 expression at nodes and internodes

a) *RN2-Gal4* and b) *RN2-Gal4>UAS-Hep^{CA}* stage 16 embryos Fas 2 immunoreactivity. Maximum projection of ventral views across two-three VNC abdominal segments. Overlaid squares represent the fixed area ROIs used for measuring the Average Integrated Density of Fas 2 signal, either at nodes (magenta) or internodes (yellow). Scale bar 10 μm.

c and d) Quantification of the Average Integrated Density of Fas 2 signal at node (c) and internode (d) positions as indicated. Nodes (magenta perimeter) (*RN2-Gal4*, (grey) n = 18; *RN2-Gal4>UAS-Hep^{CA}*, (blue) n = 32); Internodes (yellow perimeter) (*RN2-Gal4*, (grey) n = 18; *RN2-Gal4>UAS-Hep^{CA}*, (blue) n = 28). Data are presented as mean values ± SD. Parametric Student t-tests were employed. Statistically significant differences in Fas 2 levels (****, $p < 0.0001$) were detected between embryos expressing and non-expressing Hep^{CA}, both at the node and internode positions.

e) *RN2-Gal>UAS-GFP* and f) *RN2-Gal4>UAS-GFP, UAS-Hep^{CA}* stage 16 embryos Dcp 1 immunoreactivity (magenta). Maximum projection of ventral views across three VNC abdominal segments. Upon Hep^{CA} overexpression RN2 positive cells (green) become misplaced but do not die. The quantification of the number of dying cells per segment (see text) shows that apoptosis is somehow suppressed in this condition. Scale bar 10 μm.

Source data are provided as a Source Data file.

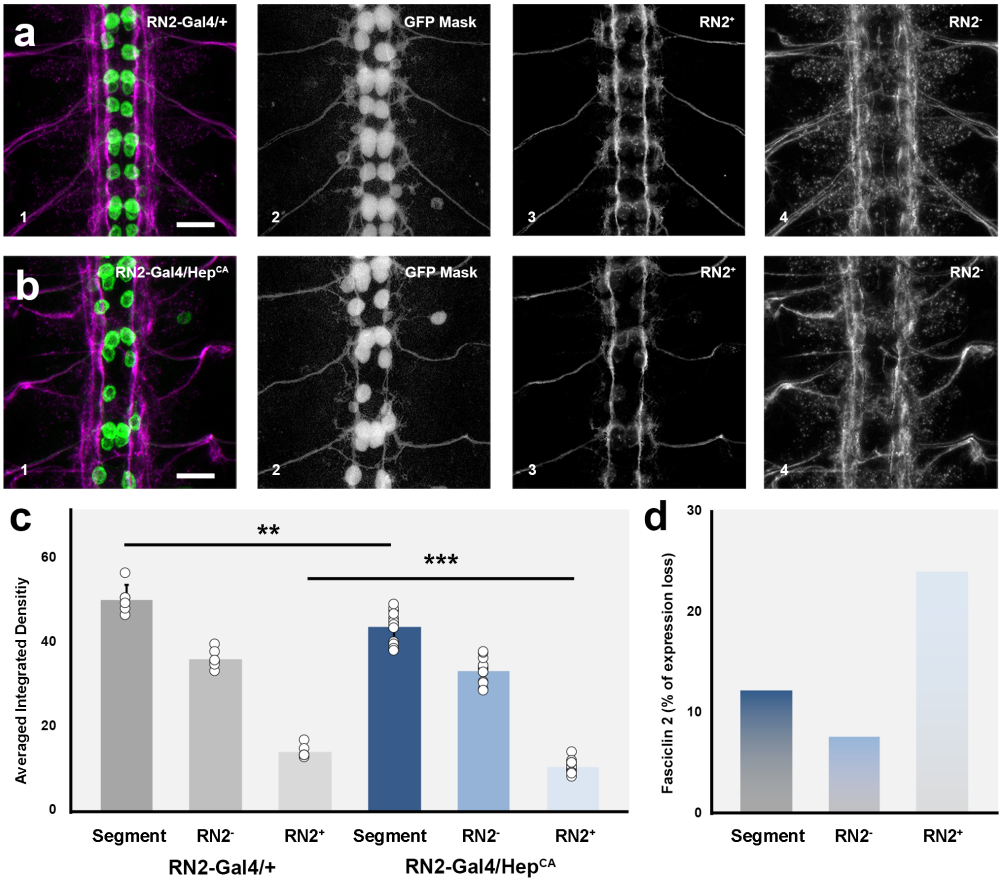
a
RN2-Gal4/+
GFP Mask
RN2+
RN2-
1
2
3
4
b
RN2-Gal4/HepCA
GFP Mask
RN2+
RN2-
1
2
3
4
c
Averaged Integrated Densitiy
60
40
20
0
**
***
Segment
RN2-
RN2+
Segment
RN2-
RN2+
RN2-Gal4/+
RN2-Gal4/HepCA
d
Fasciclin 2 (% of expression loss)
30
20
10
0
Segment
RN2-
RN2+

Supplementary Figure 10. JNK signaling autonomously modulates Fasciclin 2 expression

a) *RN2-Gal4 / +* and b) *RN2-Gal4>UAS-Hep^{CA}* stage 16 embryos Fas 2 immunoreactivity. Sum projection of ventral views across two-three VNC abdominal segments. Panels from left to right display: 1) Maximum projection of GFP (RN2-Gal4) (green) and Fas 2 (magenta) expression; 2) Sum projection of GFP binary masks (GFP signal was overexposed to retrieve the whole RN2 expression pattern and sum up regions with low RN2 expression levels); 3) Fas 2 expression associated to RN2 positive cells; and 4) Fas 2 expression associated to RN2 negative areas. Scale bar 10 μm.

c) Graphic representation of the change in Fas 2 Average Integrated Density per segment between conditions (a) (wild type controls; n = 6) and (b) (Hep^{CA} overexpressing embryos; n = 13). Data are presented as mean values ± SD. Parametric Student t-tests were employed. Statistically significant differences in Fas 2 levels were detected for whole segments (grey vs blue) (**, p = 0.0026) and for RN2 positive cells (light grey vs light blue) (***, p = 0.0003) but not for RN2 negative areas (medium gray vs medium blue) (p = 0.0557).

d) JNK hyperactivation in RN2 positive cells leads to an overall reduction of 12.6 % in Fas 2 levels in the VNC (grey/blue). This is mostly restricted to the RN2 cells, which show an autonomous reduction of Fas 2 expression of 24.8 % (light grey/light blue). Meanwhile, outside the targeted area the reduction of Fas 2 expression was not significative (7.8 %) (medium grey/medium blue).

Source data are provided as a Source Data file.

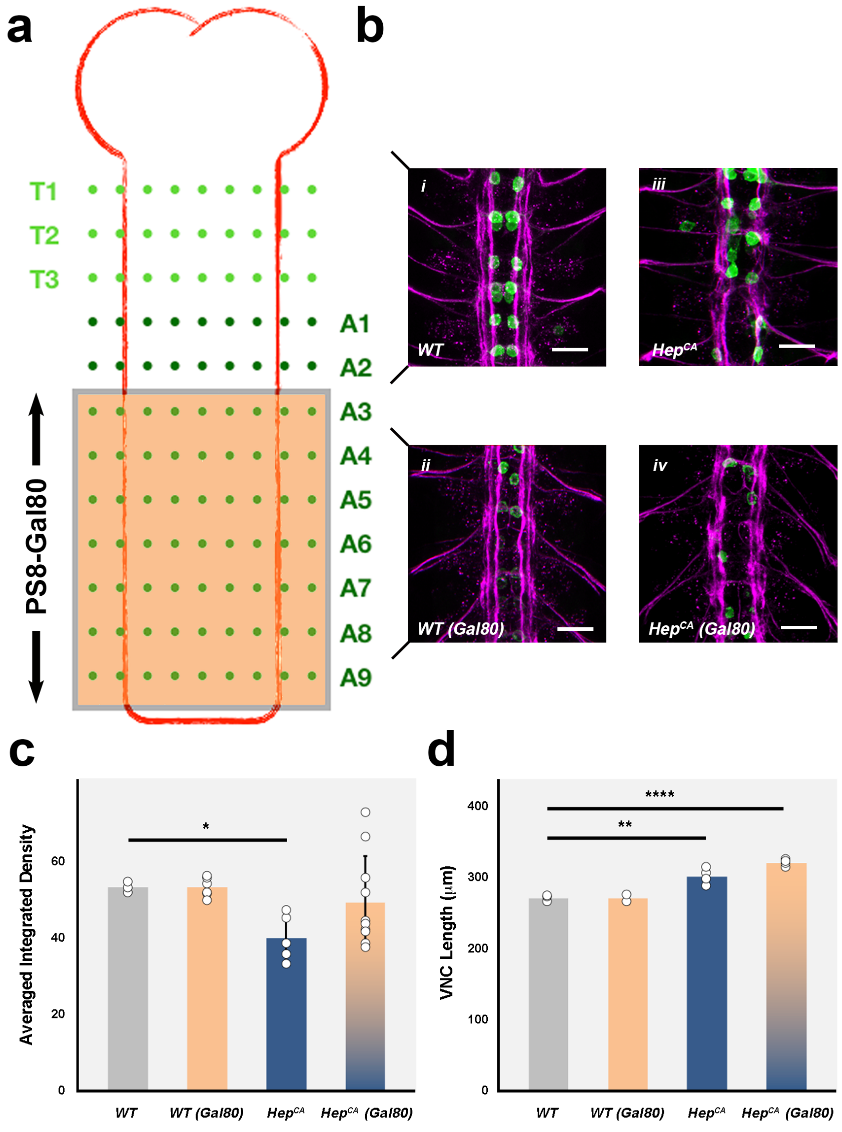

a
b
T1
T2
T3
A1
A2
A3
A4
A5
A6
A7
A8
A9
PS8-Gal80
i
WT
iii
Hep^{CA}
ii
WT (Gal80)
iv
Hep^{CA} (Gal80)
c
d
Averaged Integrated Density
0
20
40
60
*
WT
WT (Gal80)
Hep^{CA}
Hep^{CA} (Gal80)
VNC Length (μm)
0
100
200
300
400
****
**
WT
WT (Gal80)
Hep^{CA}
Hep^{CA} (Gal80)

Supplementary Figure 11. Fasciclin 2 is locally expressed under the control of JNK signaling

a) Cartoon showing a schematic view of the embryonic CNS highlighting the territory of expression of a Gal80 transgene (PS8-Gal80) under the control of the *iab3* promoter (parasegments 8 to 13) (box). Thoracic (light green) and abdominal (dark green) segments are indicated.

b) Maximum projection of ventral views across three VNC segments of *RN2-Gal4>UAS-GFP* (i and ii) and *RN2-Gal4>UAS-GFP; UAS-Hep*^{CA} (iii and iv) carrying PS8-Gal80. GFP expression is shown in green and Fas 2 in magenta. GFP is highly expressed in RN2 cells in the thoracic and anterior abdominal segments (i and iii), while its expression is strongly reduced in the posterior abdominal segments (ii and iv). Scale bar 10 µm.

c) Quantification of the Average Integrated Density of Fas 2 signal per segment. *RN2>GFP (Gal80+), T1-A2*, (grey) n = 3; *A3-A9*, (salmon) n = 6; *RN2>GFP; Hep*^{CA} *(Gal80+)*, *T1-A2*, (blue) n = 5; *A3-A9*, (blue/salmon) n = 10. Data are presented as mean values ± SD. Parametric Student t-tests were employed. Statistically significant differences in Fas 2 levels (*, p = 0.0112) were detected in areas anterior to the parasegment 8 between embryos expressing and non-expressing Hep^{CA}. No statistically significant differences were observed posteriorly (p = 0.4695). Restricting JNK hyperactivation to discrete segments confirm that Fas 2 expression was autonomously regulated by JNK.

d) Quantification of the VNC length in µm for *RN2>GFP*, (grey) n = 3; *RN2>GFP (Gal80+)*, (salmon) n = 2; *RN2>GFP; Hep*^{CA}, (blue) n = 4; *RN2>GFP; Hep*^{CA} *(Gal80+)*, (blue/salmon) n = 5. Data are presented as mean values ± SD, and parametric Student t-tests were employed. Statistically significant differences in length were detected between embryos expressing *Hep*^{CA} and controls, irrespectively of non-expressing (**, p = 0.0063) or expressing (****, p < 0.0001) Gal80. The presence of Gal80 does not affect the VNC length of controls (p = 0.9497).

Source data are provided as a Source Data file.

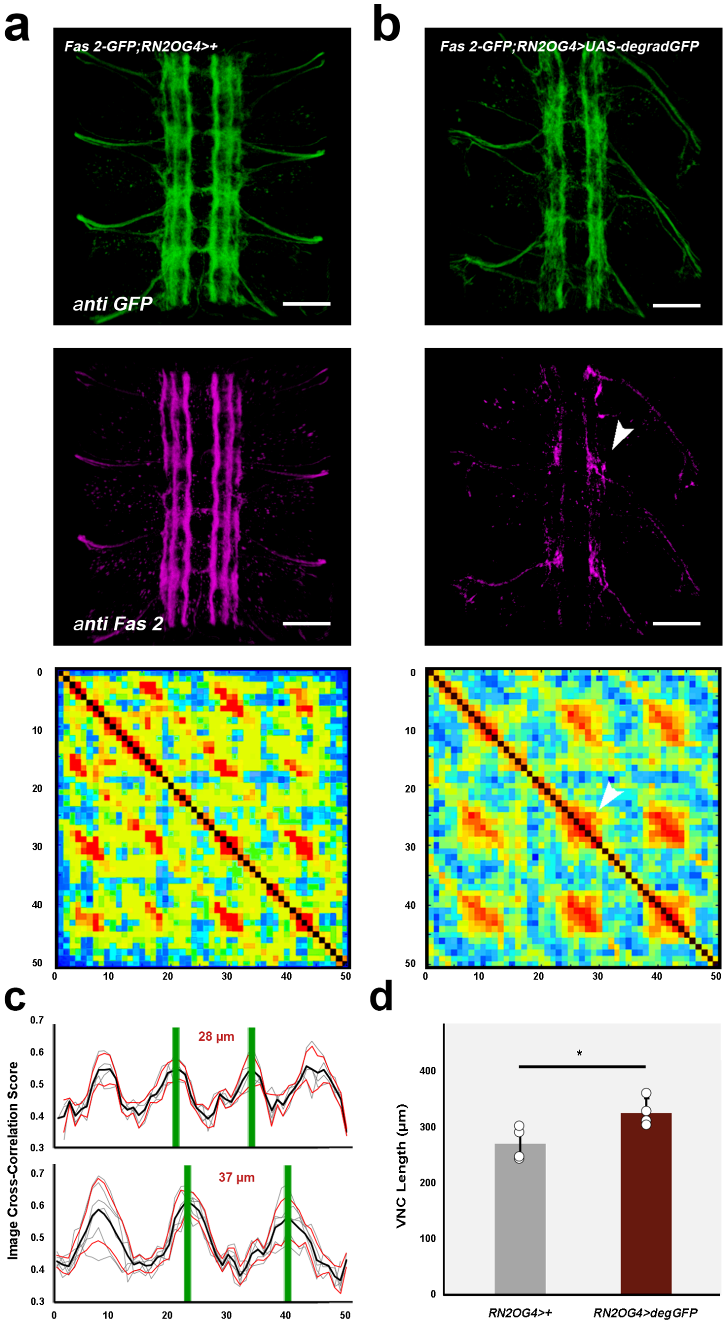
a
b
Fas 2-GFP;RN2OG4>+
Fas 2-GFP;RN2OG4>UAS-degradGFP
anti GFP
anti Fas 2
c
28 µm
37 µm
Image Cross-Correlation Score
d
VNC Length (µm)
*
RN2OG4>+
RN2OG4>degGFP

Supplementary Figure 12. Autonomous interference in Fas 2 expression in RN2 neurons leads to structural and condensation defects

a) GFP (green) and Fas 2 (magenta) immunoreactivity (sum projection of ventral views across two-three VNC abdominal segments; Scale Bar is 10 µm) and self-cross-correlation matrix (top to bottom) of the Fas 2-GFP signal in control *Fas 2-GFP; RN2OGal4>+* embryos. Scales and colormap as in Figure 1.

b) Equivalent images and data as in (a) for *Fas 2-GFP; RN2OGal4>UAS-degrad-GFP* embryos. Arrowheads point to the remnants of Fas 2 expression in the experimental embryos and to the enlarged segmental structural 3D nodes. In both cases the self cross-correlation analysis was performed on the persistant Fas 2-GFP signal. Scales and colormap as in Figure 1.

c) Image cross-correlation score along the AP axis for (a) (n = 4 embryos) and (b) (n = 6 embryos). Segmental Fas 2 3D nodes are on average at 37 µm from each other in experimental animals versus 28 µm in controls and the internodal profile loses its AP bias. The grey lines show individual profiles, black lines correspond to Average values and red lines to SD. The green bars indicate the positions of maximum correlation (3D nodes).

d) Quantification of the VNC length in µm for *Fas 2-GFP; RN2OGal4>+* (grey) (n = 4) and *Fas 2-GFP; RN2OGal4>UAS-degrad-GFP* (dark red) (n = 4) embryos. Data are presented as mean values ± SD. Parametric Student t-tests were employed. Statistically significant differences in length (*, p = 0.0305) were detected.

Source data are provided as a Source Data file.

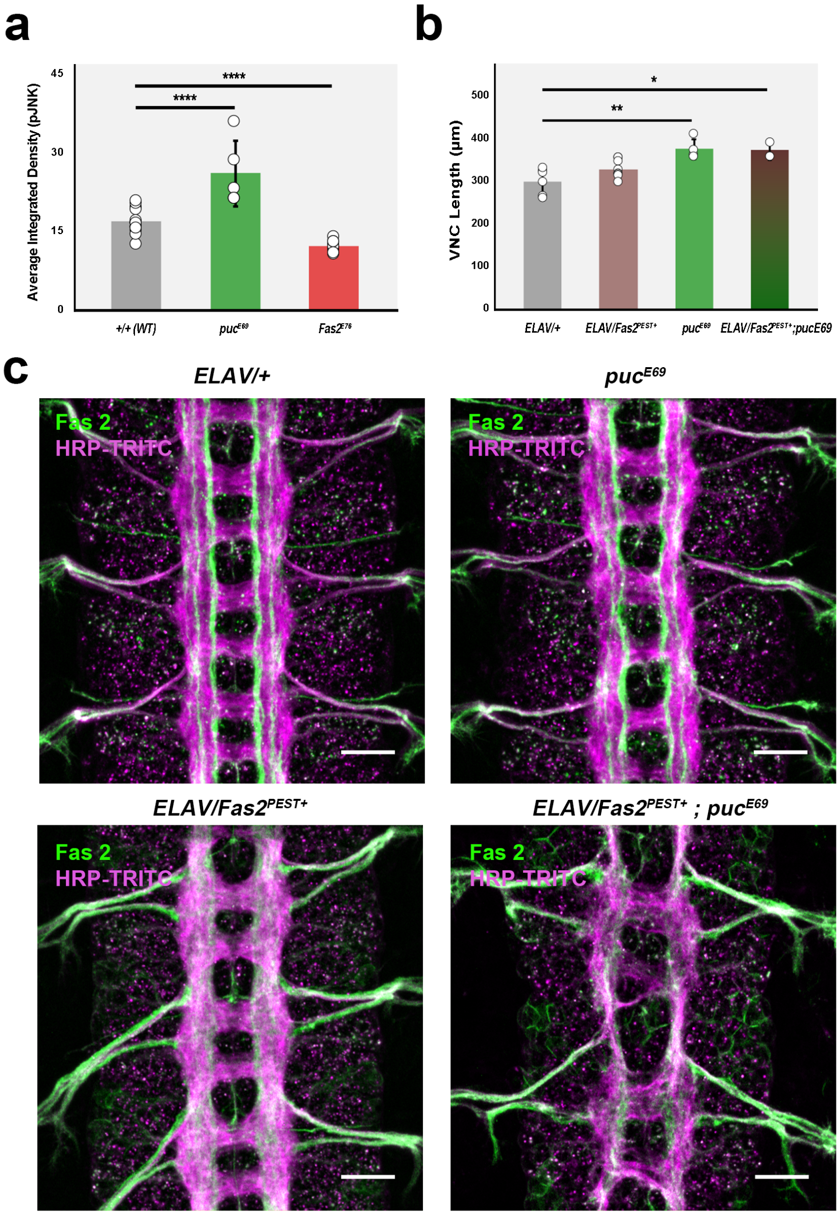
a
b
c
Average Integrated Density (pJNK)
45
30
15
0
****
****
+/+ (WT)
pucE69
Fas2E76
VNC Length (µm)
500
400
300
200
100
0
*
**
ELAV/+
ELAV/Fas2PEST+
pucE69
ELAV/Fas2PEST+;pucE69
ELAV/+
pucE69
ELAV/Fas2PEST+
ELAV/Fas2PEST+ ; pucE69
Fas 2
HRP-TRITC

Supplementary Figure 13. Fas 2 and JNK signaling interrelate but Fas 2 does not suppress the VNC structural defects of *puc*^{E69}

a) Graphic representation of the P-JNK Average Integrated Density per segment for *puc*^{E69} (green) (n = 5) and *Fas 2*^{E76} (red) (n = 13) mutants and wild type (grey) (n = 17) embryos. Data are presented as mean values ± SD. Parametric Student t-tests were employed. Statistically significant differences (****, $p < 0.0001$) in P-JNK levels were detected for whole segments between both mutant conditions and the wild type.

b) Quantification of the VNC length in µm (average and standard deviation) for each condition below (*Elav-Gal4/*+ (grey) (n = 6), *Elav-Gal4/Fas 2*$^{PEST+}$ (coral) (n = 8), *puc*$^{E\ 69}$ (green)(n = 4) and *Elav-Gal4/Fas 2*$^{PEST+}$*; puc*^{E69} (green/coral) (n = 2)) (c). Data are presented as mean values ± SD. Parametric Student t-tests were employed. Statistically significant differences in length were detected between wild type and *puc*^{E69} embryos, both in the absence (**, $p = 0.0032$) or the presence (*, $p = 0.0228$) of Fas 2^{PEST+}. The presence of Fas 2^{PEST+} does not affect the VNC length of controls ($p = 0.0593$).

c) From left to right and top to bottom, sum projections of ventral views across two-three VNC abdominal segments of *Elav-Gal4/*+, *puc*^{E69}, *Elav-Gal4/Fas 2*$^{PEST+}$ and *Elav-Gal4/Fas 2*$^{PEST+}$*; puc*^{E69} stage 16 embryos. Fas 2 and HRP immunoreactivity are shown in green and magenta respectively. Scale bar 10 µm.

Source data are provided as a Source Data file.

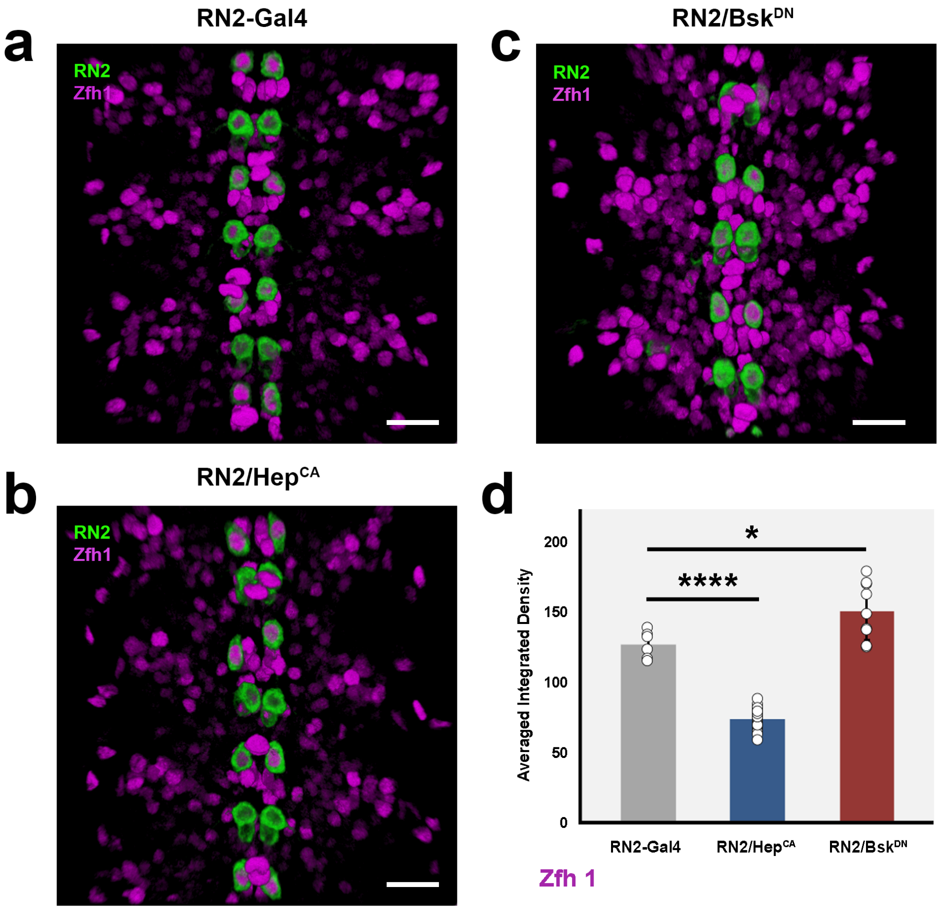
a
RN2-Gal4
RN2
Zfh1
b
RN2/Hep^CA
RN2
Zfh1
c
RN2/Bsk^DN
RN2
Zfh1
d
Averaged Integrated Density
200
150
100
50
0
*
****
RN2-Gal4
RN2/Hep^CA
RN2/Bsk^DN
Zfh 1

Supplementary Figure 14. Zfh1 levels are autonomously modulated in RN2 cells by JNK signaling

a) *RN2-Gal4 / +*, b) *RN2-Gal4>UAS- Hep*^{CA} and c) *RN2-Gal4>UAS- Bsk*^{DN} stage 16 embryos Zfh1 (magenta) immunoreactivity. Sum projection of ventral views across three VNC abdominal segments. Scale bar 10 µm.

d) Graphic representation of the reduction of Zfh1 Average Integrated Density per segment between conditions (b) (Hep^{CA} overexpressing embryos), (c) (Bsk^{DN} overexpressing embryos) and (a) (wild type controls). *RN2/+*, (n = 6); *RN2>Hep*^{CA}, (n = 22); *RN2>Bsk*^{DN}, (n = 9). Data are presented as mean values ± SD. Parametric Student t-tests were employed. Statistically significant differences in Zfh1 levels were detected for whole segments (****, $p < 0.0001$ for *RN2>Hep*^{CA} and *, $p < 0.0198$ for *RN2>Bsk*^{DN} vs *RN2/+*).

Source data are provided as a Source Data file.
