# Supplementary material for: JNK signaling in pioneer neurons organizes ventral nerve cord architecture in Drosophila embryos
Source: Nat Commun. 2023 Feb 7;14:675. doi: 10.1038/s41467-023-36388-1 (PMC9905486; doi:10.1038/s41467-023-36388-1)
Supplement: Supplementary file 3 — Description of Additional Supplementary Files [file 41467_2023_36388_MOESM3_ESM.pdf]

**Description of Additional Supplementary Files**

File Name: Supplementary Movie 1
Description: Fas 2 positive connectives
Animated 3D reconstruction of the Fas 2 positive connectives of a section of the VNC of a late embryo (Stage 17).

File Name: Supplementary Movie 2
Description: Fas 2 positive connectives of a $puc^{E69}$ embryo
Animated 3D reconstruction of the Fas 2 positive connectives of a section of the VNC of a late $puc^{E69}$ mutant embryo (Stage 17).

File Name: Supplementary Movie 3
Description: Dynamics of $puc^{E69}$ expression
Time course of $puc^{E69}$ expression from a $puc^{E69}$*I-Gal4>UAS-GFP* viable line. *puc* expression can be observed from early stage 13 in a few neurons at the midline. As development progressed, more and more neurons express *puc*, both within the VNC and at the peripheral nervous system (PNS). Scale bar is 50 µm. Anterior is to the left.

File Name: Supplementary Movie 4
Description: Minimum consensus for the segmental $puc^{E69}$ expression
Animated 3D cartoon representing a single segment of a late $puc^{E69}$ mutant embryo (Stage 17). The 18 *puc* expressing cells per hemisegment represented in the minimum consensus are shown in different colors.
